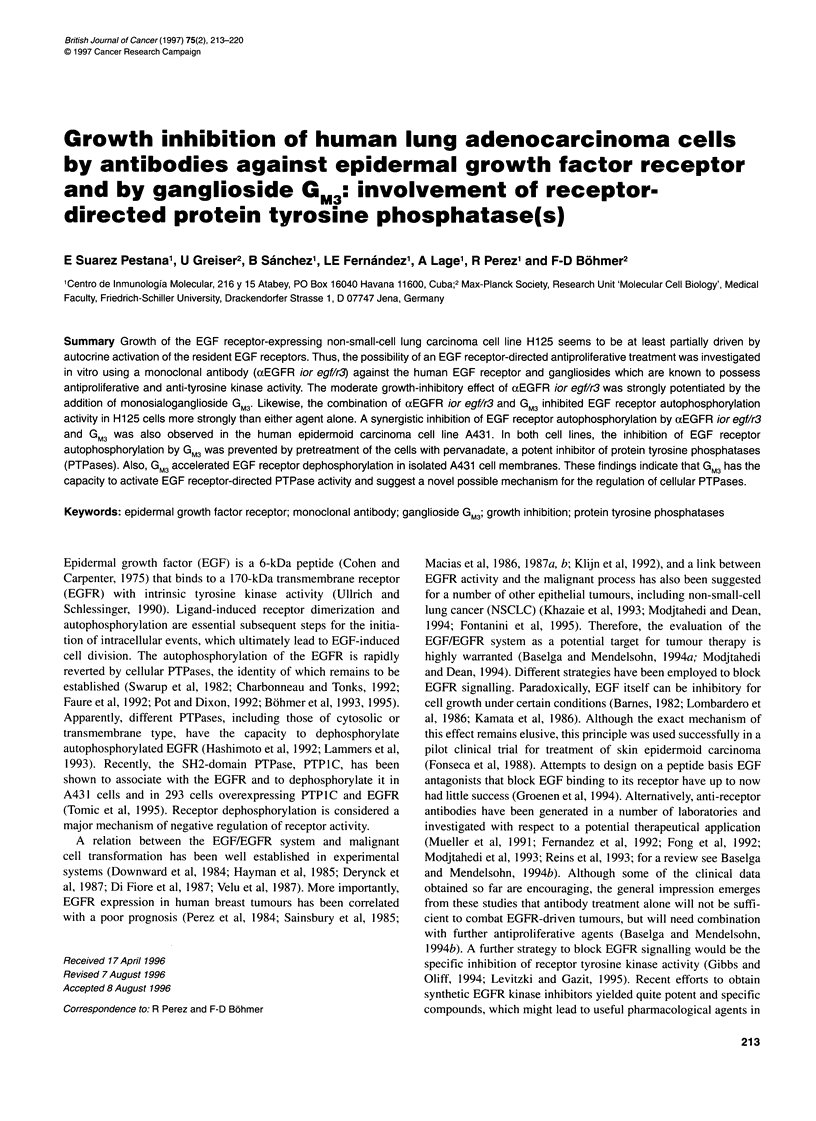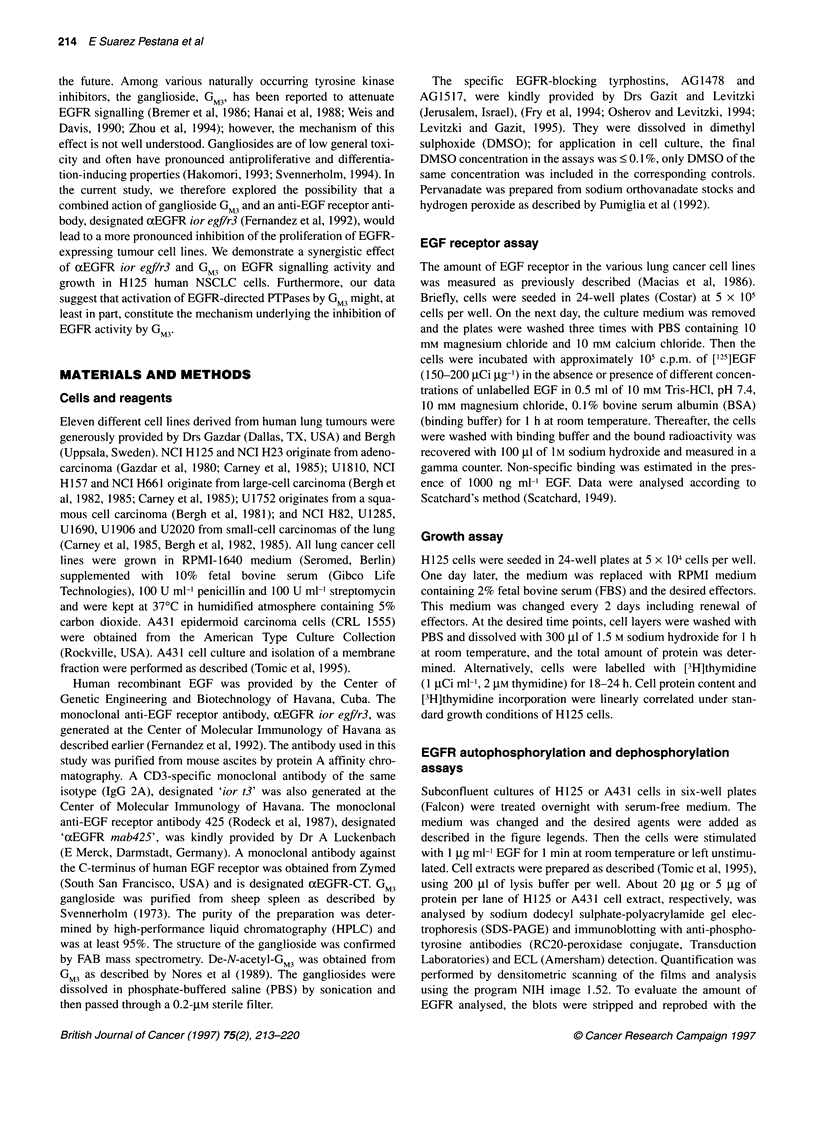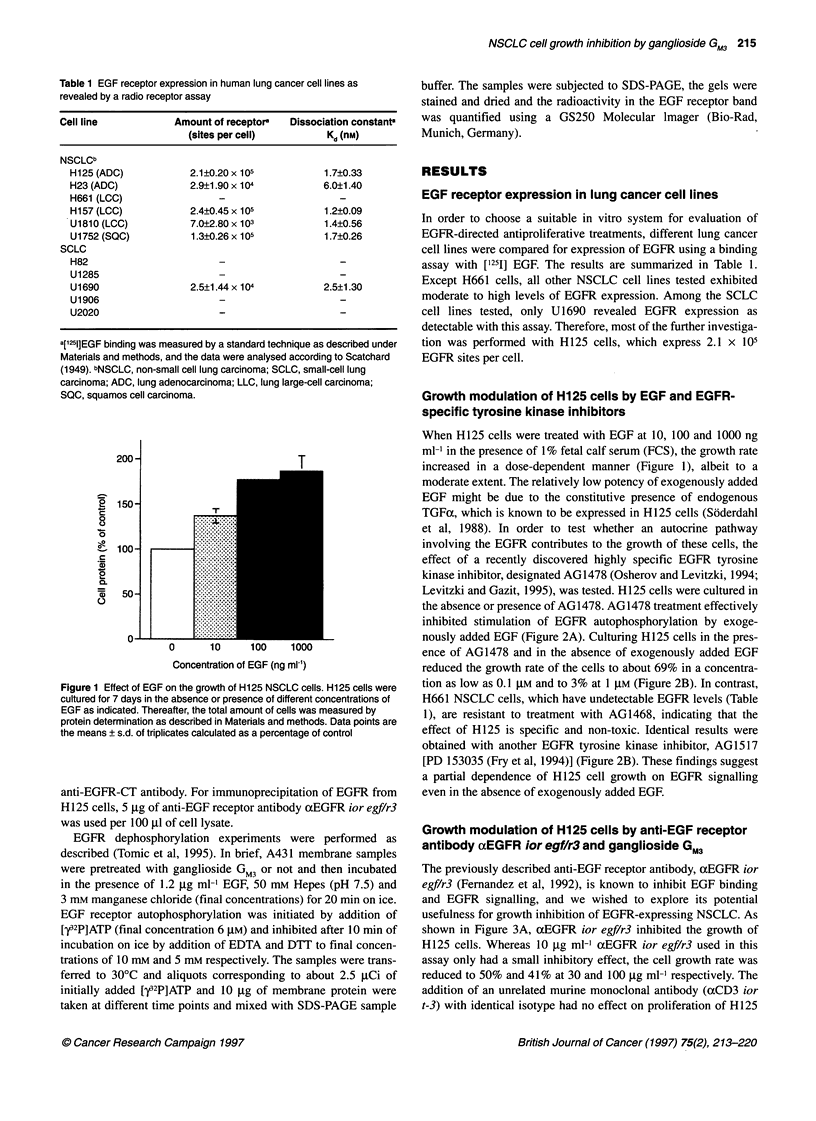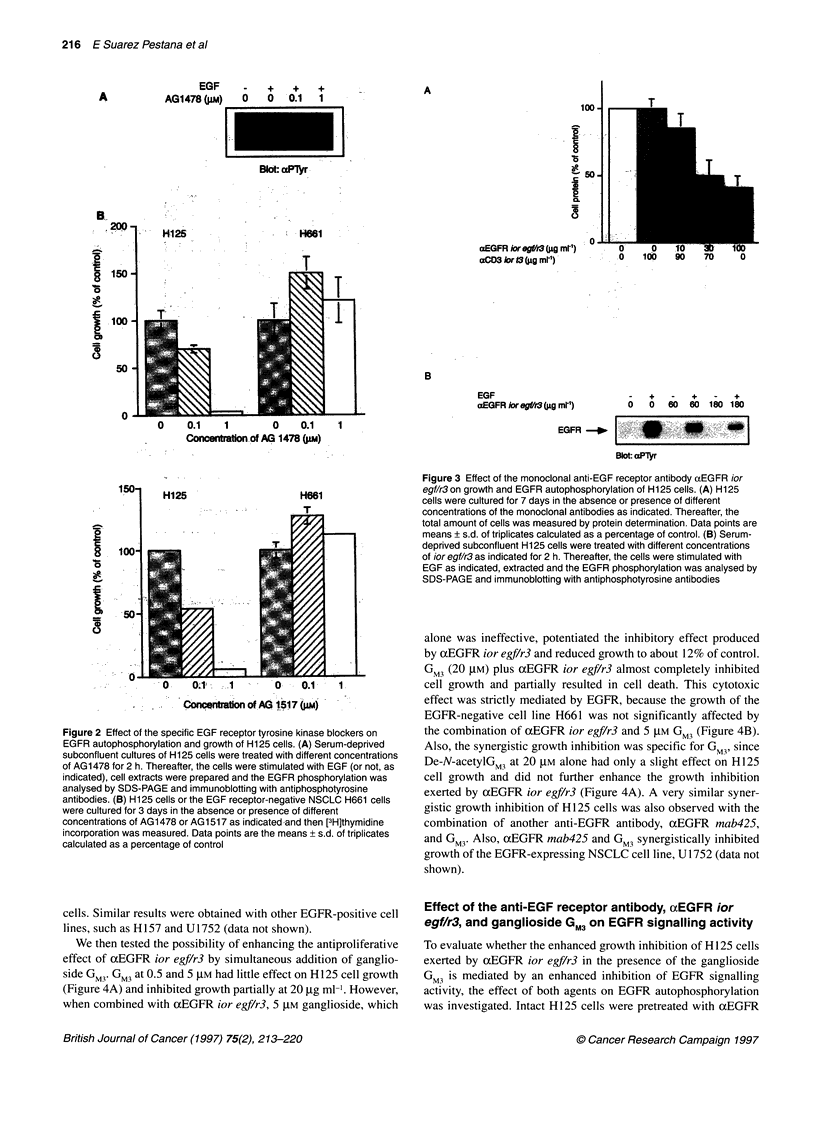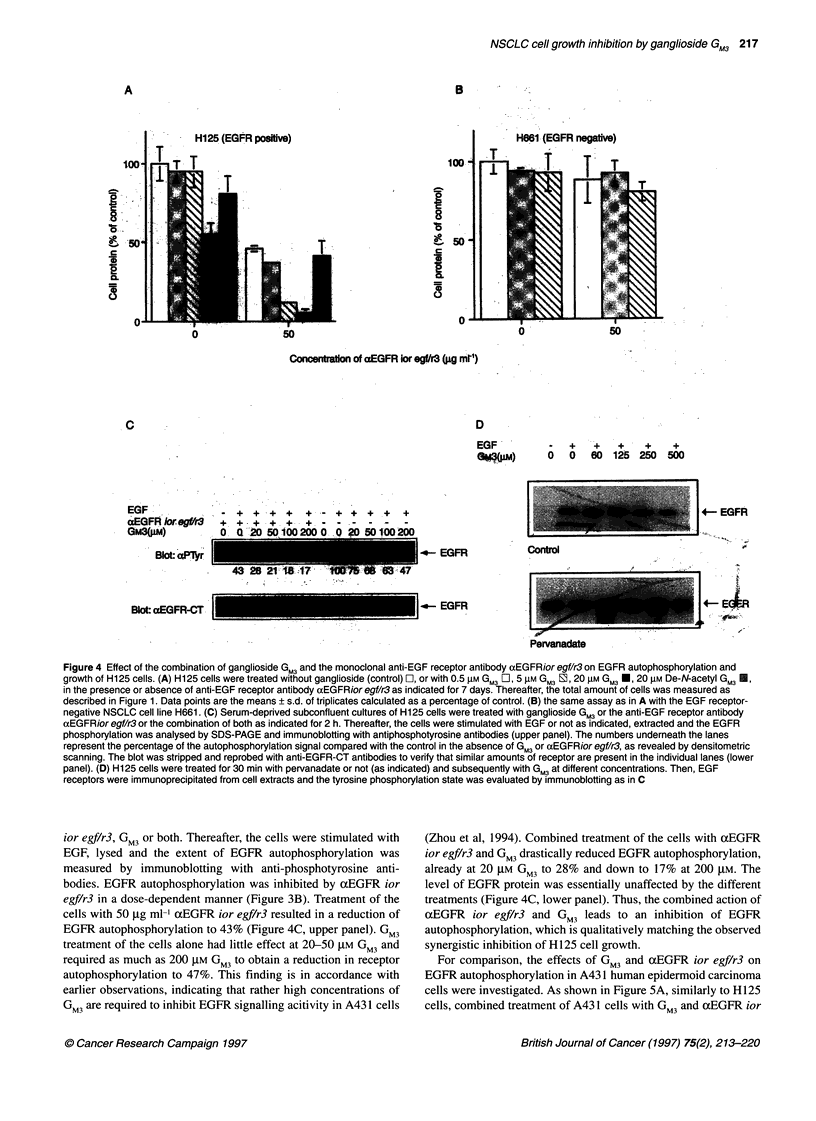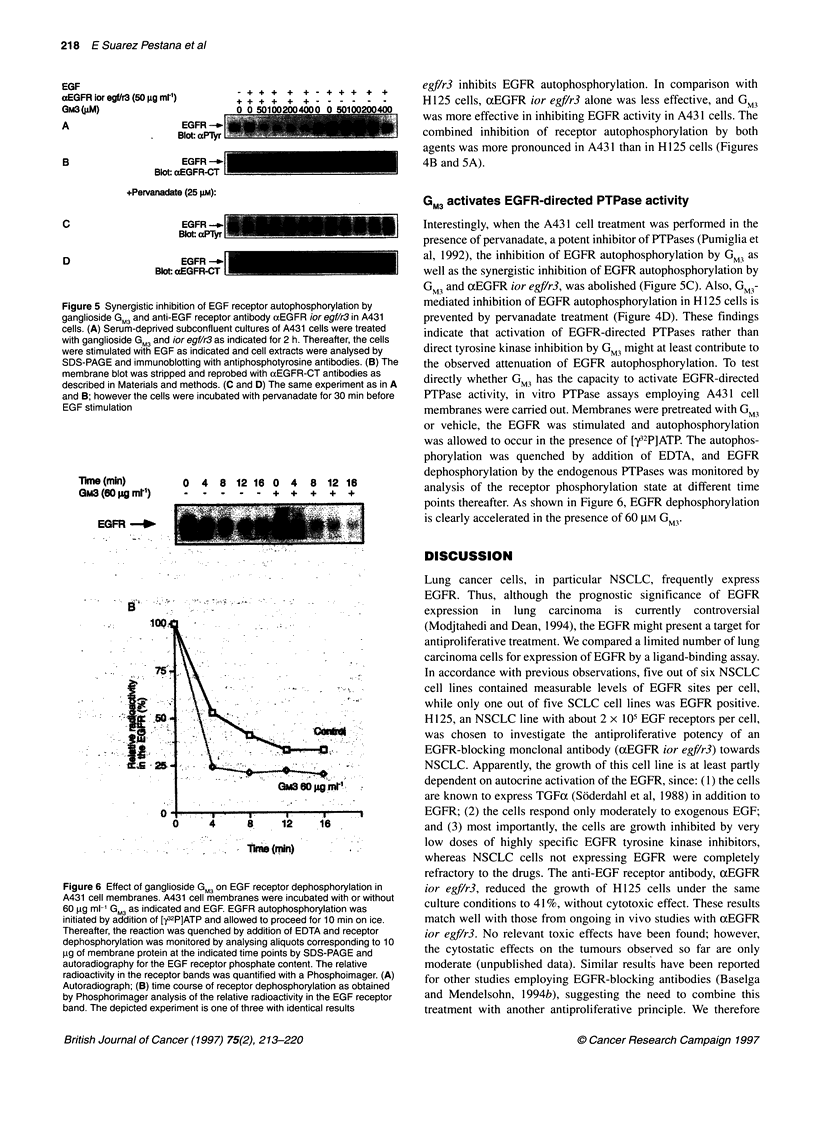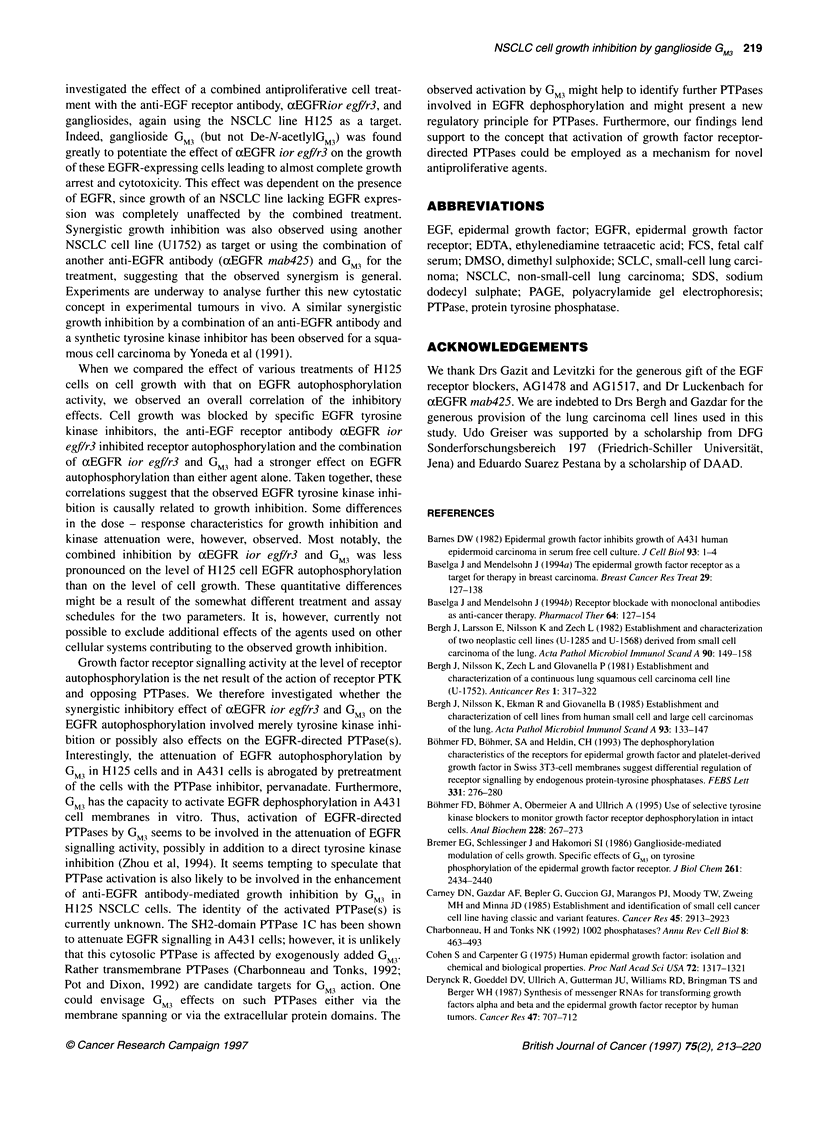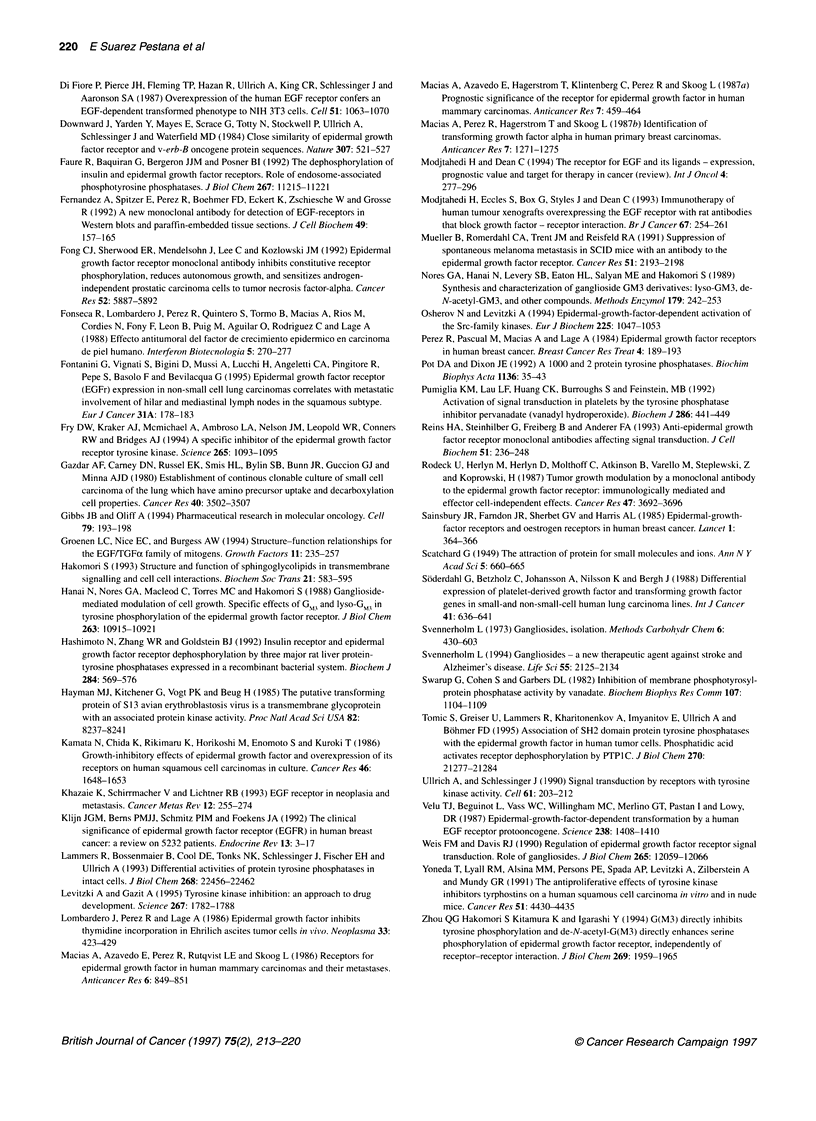# Growth inhibition of human lung adenocarcinoma cells by antibodies against epidermal growth factor receptor and by ganglioside GM3: involvement of receptor-directed protein tyrosine phosphatase(s).

**DOI:** 10.1038/bjc.1997.36

**Published:** 1997

**Authors:** E. Suarez Pestana, U. Greiser, B. Sánchez, L. E. Fernández, A. Lage, R. Perez, F. D. Böhmer

**Affiliations:** Centro de Inmunologia Molecular, Havana, Cuba.

## Abstract

**Images:**


British Journal of Cancer (1997) 75(2), 213–220
© 1997 Cancer Research Campaign

### Growth inhibition of human lung adenocarcinoma cells by antibodies against epidermal growth factor receptor and by ganglioside $G_{M3}$: involvement of receptor-directed protein tyrosine phosphatase(s)

**E Suarez Pestana[1], U Greiser[2], B Sánchez[1], LE Fernández[1], A Lage[1], R Perez[1] and F-D Böhmer[2]**

[1]Centro de Inmunología Molecular, 216 y 15 Atabey, PO Box 16040 Havana 11600, Cuba;[2] Max-Planck Society, Research Unit 'Molecular Cell Biology', Medical Faculty, Friedrich-Schiller University, Drackendorfer Strasse 1, D 07747 Jena, Germany

**Summary** Growth of the EGF receptor-expressing non-small-cell lung carcinoma cell line H125 seems to be at least partially driven by autocrine activation of the resident EGF receptors. Thus, the possibility of an EGF receptor-directed antiproliferative treatment was investigated in vitro using a monoclonal antibody (αEGFR *ior egf/r3*) against the human EGF receptor and gangliosides which are known to possess antiproliferative and anti-tyrosine kinase activity. The moderate growth-inhibitory effect of αEGFR *ior egf/r3* was strongly potentiated by the addition of monosialoganglioside $G_{M3}$. Likewise, the combination of αEGFR *ior egf/r3* and $G_{M3}$ inhibited EGF receptor autophosphorylation activity in H125 cells more strongly than either agent alone. A synergistic inhibition of EGF receptor autophosphorylation by αEGFR *ior egf/r3* and $G_{M3}$ was also observed in the human epidermoid carcinoma cell line A431. In both cell lines, the inhibition of EGF receptor autophosphorylation by $G_{M3}$ was prevented by pretreatment of the cells with pervanadate, a potent inhibitor of protein tyrosine phosphatases (PTPases). Also, $G_{M3}$ accelerated EGF receptor dephosphorylation in isolated A431 cell membranes. These findings indicate that $G_{M3}$ has the capacity to activate EGF receptor-directed PTPase activity and suggest a novel possible mechanism for the regulation of cellular PTPases.

**Keywords:** epidermal growth factor receptor; monoclonal antibody; ganglioside $G_{M3}$; growth inhibition; protein tyrosine phosphatases

Epidermal growth factor (EGF) is a 6-kDa peptide (Cohen and Carpenter, 1975) that binds to a 170-kDa transmembrane receptor (EGFR) with intrinsic tyrosine kinase activity (Ullrich and Schlessinger, 1990). Ligand-induced receptor dimerization and autophosphorylation are essential subsequent steps for the initiation of intracellular events, which ultimately lead to EGF-induced cell division. The autophosphorylation of the EGFR is rapidly reverted by cellular PTPases, the identity of which remains to be established (Swarup et al, 1982; Charbonneau and Tonks, 1992; Faure et al, 1992; Pot and Dixon, 1992; Böhmer et al, 1993, 1995). Apparently, different PTPases, including those of cytosolic or transmembrane type, have the capacity to dephosphorylate autophosphorylated EGFR (Hashimoto et al, 1992; Lammers et al, 1993). Recently, the SH2-domain PTPase, PTP1C, has been shown to associate with the EGFR and to dephosphorylate it in A431 cells and in 293 cells overexpressing PTP1C and EGFR (Tomic et al, 1995). Receptor dephosphorylation is considered a major mechanism of negative regulation of receptor activity.

A relation between the EGF/EGFR system and malignant cell transformation has been well established in experimental systems (Downward et al, 1984; Hayman et al, 1985; Derynck et al, 1987; Di Fiore et al, 1987; Velu et al, 1987). More importantly, EGFR expression in human breast tumours has been correlated with a poor prognosis (Perez et al, 1984; Sainsbury et al, 1985; Macias et al, 1986, 1987*a, b*; Klijn et al, 1992), and a link between EGFR activity and the malignant process has also been suggested for a number of other epithelial tumours, including non-small-cell lung cancer (NSCLC) (Khazaie et al, 1993; Modjtahedi and Dean, 1994; Fontanini et al, 1995). Therefore, the evaluation of the EGF/EGFR system as a potential target for tumour therapy is highly warranted (Baselga and Mendelsohn, 1994*a;* Modjtahedi and Dean, 1994). Different strategies have been employed to block EGFR signalling. Paradoxically, EGF itself can be inhibitory for cell growth under certain conditions (Barnes, 1982; Lombardero et al, 1986; Kamata et al, 1986). Although the exact mechanism of this effect remains elusive, this principle was used successfully in a pilot clinical trial for treatment of skin epidermoid carcinoma (Fonseca et al, 1988). Attempts to design on a peptide basis EGF antagonists that block EGF binding to its receptor have up to now had little success (Groenen et al, 1994). Alternatively, anti-receptor antibodies have been generated in a number of laboratories and investigated with respect to a potential therapeutical application (Mueller et al, 1991; Fernandez et al, 1992; Fong et al, 1992; Modjtahedi et al, 1993; Reins et al, 1993; for a review see Baselga and Mendelsohn, 1994*b*). Although some of the clinical data obtained so far are encouraging, the general impression emerges from these studies that antibody treatment alone will not be sufficient to combat EGFR-driven tumours, but will need combination with further antiproliferative agents (Baselga and Mendelsohn, 1994*b*). A further strategy to block EGFR signalling would be the specific inhibition of receptor tyrosine kinase activity (Gibbs and Oliff, 1994; Levitzki and Gazit, 1995). Recent efforts to obtain synthetic EGFR kinase inhibitors yielded quite potent and specific compounds, which might lead to useful pharmacological agents in

*Received 17 April 1996*
*Revised 7 August 1996*
*Accepted 8 August 1996*

*Correspondence to:* R Perez and F-D Böhmer the future. Among various naturally occurring tyrosine kinase inhibitors, the ganglioside, $G_{M3}$, has been reported to attenuate EGFR signalling (Bremer et al, 1986; Hanai et al, 1988; Weis and Davis, 1990; Zhou et al, 1994); however, the mechanism of this effect is not well understood. Gangliosides are of low general toxicity and often have pronounced antiproliferative and differentiation-inducing properties (Hakomori, 1993; Svennerholm, 1994). In the current study, we therefore explored the possibility that a combined action of ganglioside $G_{M3}$ and an anti-EGF receptor antibody, designated αEGFR *ior egf/r3* (Fernandez et al, 1992), would lead to a more pronounced inhibition of the proliferation of EGFR-expressing tumour cell lines. We demonstrate a synergistic effect of αEGFR *ior egf/r3* and $G_{M3}$ on EGFR signalling activity and growth in H125 human NSCLC cells. Furthermore, our data suggest that activation of EGFR-directed PTPases by $G_{M3}$ might, at least in part, constitute the mechanism underlying the inhibition of EGFR activity by $G_{M3}$.

### MATERIALS AND METHODS

### Cells and reagents

Eleven different cell lines derived from human lung tumours were generously provided by Drs Gazdar (Dallas, TX, USA) and Bergh (Uppsala, Sweden). NCI H125 and NCI H23 originate from adenocarcinoma (Gazdar et al, 1980; Carney et al, 1985); U1810, NCI H157 and NCI H661 originate from large-cell carcinoma (Bergh et al, 1982, 1985; Carney et al, 1985); U1752 originates from a squamous cell carcinoma (Bergh et al, 1981); and NCI H82, U1285, U1690, U1906 and U2020 from small-cell carcinomas of the lung (Carney et al, 1985, Bergh et al, 1982, 1985). All lung cancer cell lines were grown in RPMI-1640 medium (Seromed, Berlin) supplemented with 10% fetal bovine serum (Gibco Life Technologies), 100 U $ml^{-1}$ penicillin and 100 U $ml^{-1}$ streptomycin and were kept at 37°C in humidified atmosphere containing 5% carbon dioxide. A431 epidermoid carcinoma cells (CRL 1555) were obtained from the American Type Culture Collection (Rockville, USA). A431 cell culture and isolation of a membrane fraction were performed as described (Tomic et al, 1995).

Human recombinant EGF was provided by the Center of Genetic Engineering and Biotechnology of Havana, Cuba. The monoclonal anti-EGF receptor antibody, αEGFR *ior egf/r3*, was generated at the Center of Molecular Immunology of Havana as described earlier (Fernandez et al, 1992). The antibody used in this study was purified from mouse ascites by protein A affinity chromatography. A CD3-specific monoclonal antibody of the same isotype (IgG 2A), designated '*ior t3*' was also generated at the Center of Molecular Immunology of Havana. The monoclonal anti-EGF receptor antibody 425 (Rodeck et al, 1987), designated 'αEGFR *mab425*', was kindly provided by Dr A Luckenbach (E Merck, Darmstadt, Germany). A monoclonal antibody against the C-terminus of human EGF receptor was obtained from Zymed (South San Francisco, USA) and is designated αEGFR-CT. $G_{M3}$ gangloside was purified from sheep spleen as described by Svennerholm (1973). The purity of the preparation was determined by high-performance liquid chromatography (HPLC) and was at least 95%. The structure of the ganglioside was confirmed by FAB mass spectrometry. De-*N*-acetyl-$G_{M3}$ was obtained from $G_{M3}$ as described by Nores et al (1989). The gangliosides were dissolved in phosphate-buffered saline (PBS) by sonication and then passed through a 0.2-μM sterile filter.

The specific EGFR-blocking tyrphostins, AG1478 and AG1517, were kindly provided by Drs Gazit and Levitzki (Jerusalem, Israel), (Fry et al, 1994; Osherov and Levitzki, 1994; Levitzki and Gazit, 1995). They were dissolved in dimethyl sulphoxide (DMSO); for application in cell culture, the final DMSO concentration in the assays was ≤ 0.1%, only DMSO of the same concentration was included in the corresponding controls. Pervanadate was prepared from sodium orthovanadate stocks and hydrogen peroxide as described by Pumiglia et al (1992).

### EGF receptor assay

The amount of EGF receptor in the various lung cancer cell lines was measured as previously described (Macias et al, 1986). Briefly, cells were seeded in 24-well plates (Costar) at $5 \times 10^5$ cells per well. On the next day, the culture medium was removed and the plates were washed three times with PBS containing 10 mM magnesium chloride and 10 mM calcium chloride. Then the cells were incubated with approximately $10^5$ c.p.m. of [$^{125}$I]EGF (150–200 μCi $\mu g^{-1}$) in the absence or presence of different concentrations of unlabelled EGF in 0.5 ml of 10 mM Tris-HCl, pH 7.4, 10 mM magnesium chloride, 0.1% bovine serum albumin (BSA) (binding buffer) for 1 h at room temperature. Thereafter, the cells were washed with binding buffer and the bound radioactivity was recovered with 100 μl of 1M sodium hydroxide and measured in a gamma counter. Non-specific binding was estimated in the presence of 1000 ng $ml^{-1}$ EGF. Data were analysed according to Scatchard's method (Scatchard, 1949).

### Growth assay

H125 cells were seeded in 24-well plates at $5 \times 10^4$ cells per well. One day later, the medium was replaced with RPMI medium containing 2% fetal bovine serum (FBS) and the desired effectors. This medium was changed every 2 days including renewal of effectors. At the desired time points, cell layers were washed with PBS and dissolved with 300 μl of 1.5 M sodium hydroxide for 1 h at room temperature, and the total amount of protein was determined. Alternatively, cells were labelled with [$^3$H]thymidine (1 μCi $ml^{-1}$, 2 μM thymidine) for 18–24 h. Cell protein content and [$^3$H]thymidine incorporation were linearly correlated under standard growth conditions of H125 cells.

### EGFR autophosphorylation and dephosphorylation assays

Subconfluent cultures of H125 or A431 cells in six-well plates (Falcon) were treated overnight with serum-free medium. The medium was changed and the desired agents were added as described in the figure legends. Then the cells were stimulated with 1 μg $ml^{-1}$ EGF for 1 min at room temperature or left unstimulated. Cell extracts were prepared as described (Tomic et al, 1995), using 200 μl of lysis buffer per well. About 20 μg or 5 μg of protein per lane of H125 or A431 cell extract, respectively, was analysed by sodium dodecyl sulphate-polyacrylamide gel electrophoresis (SDS-PAGE) and immunoblotting with anti-phosphotyrosine antibodies (RC20-peroxidase conjugate, Transduction Laboratories) and ECL (Amersham) detection. Quantification was performed by densitometric scanning of the films and analysis using the program NIH image 1.52. To evaluate the amount of EGFR analysed, the blots were stripped and reprobed with the

 © Cancer Research Campaign 1997

**Table 1** EGF receptor expression in human lung cancer cell lines as revealed by a radio receptor assay

| Cell line | Amount of receptor[a] (sites per cell) | Dissociation constant[a] $K_d$ (nM) |
|---|---|---|
| NSCLC[b] | | |
| H125 (ADC) | 2.1±0.20 × $10^5$ | 1.7±0.33 |
| H23 (ADC) | 2.9±1.90 × $10^4$ | 6.0±1.40 |
| H661 (LCC) | – | – |
| H157 (LCC) | 2.4±0.45 × $10^5$ | 1.2±0.09 |
| U1810 (LCC) | 7.0±2.80 × $10^3$ | 1.4±0.56 |
| U1752 (SQC) | 1.3±0.26 × $10^5$ | 1.7±0.26 |
| SCLC | | |
| H82 | – | – |
| U1285 | – | – |
| U1690 | 2.5±1.44 × $10^4$ | 2.5±1.30 |
| U1906 | – | – |
| U2020 | – | – |

[a][$^{125}$I]EGF binding was measured by a standard technique as described under Materials and methods, and the data were analysed according to Scatchard (1949). [b]NSCLC, non-small cell lung carcinoma; SCLC, small-cell lung carcinoma; ADC, lung adenocarcinoma; LLC, lung large-cell carcinoma; SQC, squamos cell carcinoma.

EGFR dephosphorylation experiments were performed as described (Tomic et al, 1995). In brief, A431 membrane samples were pretreated with ganglioside $G_{M3}$ or not and then incubated in the presence of 1.2 μg ml$^{-1}$ EGF, 50 mM Hepes (pH 7.5) and 3 mM manganese chloride (final concentrations) for 20 min on ice. EGF receptor autophosphorylation was initiated by addition of [γ$^{32}$P]ATP (final concentration 6 μM) and inhibited after 10 min of incubation on ice by addition of EDTA and DTT to final concentrations of 10 mM and 5 mM respectively. The samples were transferred to 30°C and aliquots corresponding to about 2.5 μCi of initially added [γ$^{32}$P]ATP and 10 μg of membrane protein were taken at different time points and mixed with SDS-PAGE sample buffer. The samples were subjected to SDS-PAGE, the gels were stained and dried and the radioactivity in the EGF receptor band was quantified using a GS250 Molecular lmager (Bio-Rad, Munich, Germany).

### RESULTS

### EGF receptor expression in lung cancer cell lines

In order to choose a suitable in vitro system for evaluation of EGFR-directed antiproliferative treatments, different lung cancer cell lines were compared for expression of EGFR using a binding assay with [$^{125}$I] EGF. The results are summarized in Table 1. Except H661 cells, all other NSCLC cell lines tested exhibited moderate to high levels of EGFR expression. Among the SCLC cell lines tested, only U1690 revealed EGFR expression as detectable with this assay. Therefore, most of the further investigation was performed with H125 cells, which express 2.1 × $10^5$ EGFR sites per cell.

### Growth modulation of H125 cells by EGF and EGFR-specific tyrosine kinase inhibitors

When H125 cells were treated with EGF at 10, 100 and 1000 ng ml$^{-1}$ in the presence of 1% fetal calf serum (FCS), the growth rate increased in a dose-dependent manner (Figure 1), albeit to a moderate extent. The relatively low potency of exogenously added EGF might be due to the constitutive presence of endogenous TGFα, which is known to be expressed in H125 cells (Söderdahl et al, 1988). In order to test whether an autocrine pathway involving the EGFR contributes to the growth of these cells, the effect of a recently discovered highly specific EGFR tyrosine kinase inhibitor, designated AG1478 (Osherov and Levitzki, 1994; Levitzki and Gazit, 1995), was tested. H125 cells were cultured in the absence or presence of AG1478. AG1478 treatment effectively inhibited stimulation of EGFR autophosphorylation by exogenously added EGF (Figure 2A). Culturing H125 cells in the presence of AG1478 and in the absence of exogenously added EGF reduced the growth rate of the cells to about 69% in a concentration as low as 0.1 μM and to 3% at 1 μM (Figure 2B). In contrast, H661 NSCLC cells, which have undetectable EGFR levels (Table 1), are resistant to treatment with AG1468, indicating that the effect of H125 is specific and non-toxic. Identical results were obtained with another EGFR tyrosine kinase inhibitor, AG1517 [PD 153035 (Fry et al, 1994)] (Figure 2B). These findings suggest a partial dependence of H125 cell growth on EGFR signalling even in the absence of exogenously added EGF.

### Growth modulation of H125 cells by anti-EGF receptor antibody αEGFR *ior egf/r3* and ganglioside $G_{M3}$

The previously described anti-EGF receptor antibody, αEGFR *ior egf/r3* (Fernandez et al, 1992), is known to inhibit EGF binding and EGFR signalling, and we wished to explore its potential usefulness for growth inhibition of EGFR-expressing NSCLC. As shown in Figure 3A, αEGFR *ior egf/r3* inhibited the growth of H125 cells. Whereas 10 μg ml$^{-1}$ αEGFR *ior egf/r3* used in this assay only had a small inhibitory effect, the cell growth rate was reduced to 50% and 41% at 30 and 100 μg ml$^{-1}$ respectively. The addition of an unrelated murine monoclonal antibody (αCD3 *ior t-3*) with identical isotype had no effect on proliferation of H125

© Cancer Research Campaign 1997

**Figure 2** Effect of the specific EGF receptor tyrosine kinase blockers on EGFR autophosphorylation and growth of H125 cells. (**A**) Serum-deprived subconfluent cultures of H125 cells were treated with different concentrations of AG1478 for 2 h. Thereafter, the cells were stimulated with EGF (or not, as indicated), cell extracts were prepared and the EGFR phosphorylation was analysed by SDS-PAGE and immunoblotting with antiphosphotyrosine antibodies. (**B**) H125 cells or the EGF receptor-negative NSCLC H661 cells were cultured for 3 days in the absence or presence of different concentrations of AG1478 or AG1517 as indicated and then [$^3$H]thymidine incorporation was measured. Data points are the means ± s.d. of triplicates calculated as a percentage of control

**Figure 3** Effect of the monoclonal anti-EGF receptor antibody αEGFR *ior egf/r3* on growth and EGFR autophosphorylation of H125 cells. (**A**) H125 cells were cultured for 7 days in the absence or presence of different concentrations of the monoclonal antibodies as indicated. Thereafter, the total amount of cells was measured by protein determination. Data points are means ± s.d. of triplicates calculated as a percentage of control. (**B**) Serum-deprived subconfluent H125 cells were treated with different concentrations of *ior egf/r3* as indicated for 2 h. Thereafter, the cells were stimulated with EGF as indicated, extracted and the EGFR phosphorylation was analysed by SDS-PAGE and immunoblotting with antiphosphotyrosine antibodies cells. Similar results were obtained with other EGFR-positive cell lines, such as H157 and U1752 (data not shown).

We then tested the possibility of enhancing the antiproliferative effect of αEGFR *ior egf/r3* by simultaneous addition of ganglioside $G_{M3}$. $G_{M3}$ at 0.5 and 5 μM had little effect on H125 cell growth (Figure 4A) and inhibited growth partially at 20 μg ml$^{-1}$. However, when combined with αEGFR *ior egf/r3*, 5 μM ganglioside, which alone was ineffective, potentiated the inhibitory effect produced by αEGFR *ior egf/r3* and reduced growth to about 12% of control. $G_{M3}$ (20 μM) plus αEGFR *ior egf/r3* almost completely inhibited cell growth and partially resulted in cell death. This cytotoxic effect was strictly mediated by EGFR, because the growth of the EGFR-negative cell line H661 was not significantly affected by the combination of αEGFR *ior egf/r3* and 5 μM $G_{M3}$ (Figure 4B). Also, the synergistic growth inhibition was specific for $G_{M3}$, since De-*N*-acetyl$G_{M3}$ at 20 μM alone had only a slight effect on H125 cell growth and did not further enhance the growth inhibition exerted by αEGFR *ior egf/r3* (Figure 4A). A very similar synergistic growth inhibition of H125 cells was also observed with the combination of another anti-EGFR antibody, αEGFR *mab425*, and $G_{M3}$. Also, αEGFR *mab425* and $G_{M3}$ synergistically inhibited growth of the EGFR-expressing NSCLC cell line, U1752 (data not shown).

### Effect of the anti-EGF receptor antibody, αEGFR *ior egf/r3*, and ganglioside $G_{M3}$ on EGFR signalling activity

To evaluate whether the enhanced growth inhibition of H125 cells exerted by αEGFR *ior egf/r3* in the presence of the ganglioside $G_{M3}$ is mediated by an enhanced inhibition of EGFR signalling activity, the effect of both agents on EGFR autophosphorylation was investigated. Intact H125 cells were pretreated with αEGFR

© Cancer Research Campaign 1997

**Figure 4** Effect of the combination of ganglioside $G_{M3}$ and the monoclonal anti-EGF receptor antibody αEGFR*ior egf/r3* on EGFR autophosphorylation and growth of H125 cells. (**A**) H125 cells were treated without ganglioside (control) □, or with 0.5 μM $G_{M3}$ □, 5 μM $G_{M3}$ ▨, 20 μM $G_{M3}$ ■, 20 μM De-*N*-acetyl $G_{M3}$ ▩, in the presence or absence of anti-EGF receptor antibody αEGFR*ior egf/r3* as indicated for 7 days. Thereafter, the total amount of cells was measured as described in Figure 1. Data points are the means ± s.d. of triplicates calculated as a percentage of control. (**B**) the same assay as in **A** with the EGF receptor-negative NSCLC cell line H661. (**C**) Serum-deprived subconfluent cultures of H125 cells were treated with ganglioside $G_{M3}$ or the anti-EGF receptor antibody αEGFR*ior egf/r3* or the combination of both as indicated for 2 h. Thereafter, the cells were stimulated with EGF or not as indicated, extracted and the EGFR phosphorylation was analysed by SDS-PAGE and immunoblotting with antiphosphotyrosine antibodies (upper panel). The numbers underneath the lanes represent the percentage of the autophosphorylation signal compared with the control in the absence of $G_{M3}$ or αEGFR*ior egf/r3*, as revealed by densitometric scanning. The blot was stripped and reprobed with anti-EGFR-CT antibodies to verify that similar amounts of receptor are present in the individual lanes (lower panel). (**D**) H125 cells were treated for 30 min with pervanadate or not (as indicated) and subsequently with $G_{M3}$ at different concentrations. Then, EGF receptors were immunoprecipitated from cell extracts and the tyrosine phosphorylation state was evaluated by immunoblotting as in **C**

*ior egf/r3*, $G_{M3}$ or both. Thereafter, the cells were stimulated with EGF, lysed and the extent of EGFR autophosphorylation was measured by immunoblotting with anti-phosphotyrosine antibodies. EGFR autophosphorylation was inhibited by αEGFR *ior egf/r3* in a dose-dependent manner (Figure 3B). Treatment of the cells with 50 μg ml⁻¹ αEGFR *ior egf/r3* resulted in a reduction of EGFR autophosphorylation to 43% (Figure 4C, upper panel). $G_{M3}$ treatment of the cells alone had little effect at 20–50 μM $G_{M3}$ and required as much as 200 μM $G_{M3}$ to obtain a reduction in receptor autophosphorylation to 47%. This finding is in accordance with earlier observations, indicating that rather high concentrations of $G_{M3}$ are required to inhibit EGFR signalling acitivity in A431 cells (Zhou et al, 1994). Combined treatment of the cells with αEGFR *ior egf/r3* and $G_{M3}$ drastically reduced EGFR autophosphorylation, already at 20 μM $G_{M3}$ to 28% and down to 17% at 200 μM. The level of EGFR protein was essentially unaffected by the different treatments (Figure 4C, lower panel). Thus, the combined action of αEGFR *ior egf/r3* and $G_{M3}$ leads to an inhibition of EGFR autophosphorylation, which is qualitatively matching the observed synergistic inhibition of H125 cell growth.

For comparison, the effects of $G_{M3}$ and αEGFR *ior egf/r3* on EGFR autophosphorylation in A431 human epidermoid carcinoma cells were investigated. As shown in Figure 5A, similarly to H125 cells, combined treatment of A431 cells with $G_{M3}$ and αEGFR *ior*

© Cancer Research Campaign 1997 

**Figure 5** Synergistic inhibition of EGF receptor autophosphorylation by ganglioside $G_{M3}$ and anti-EGF receptor antibody αEGFR *ior egf/r3* in A431 cells. (**A**) Serum-deprived subconfluent cultures of A431 cells were treated with ganglioside $G_{M3}$ and *ior egf/r3* as indicated for 2 h. Thereafter, the cells were stimulated with EGF as indicated and cell extracts were analysed by SDS-PAGE and immunoblotting with antiphosphotyrosine antibodies. (**B**) The membrane blot was stripped and reprobed with αEGFR-CT antibodies as described in Materials and methods. (**C** and **D**) The same experiment as in **A** and **B**; however the cells were incubated with pervanadate for 30 min before EGF stimulation

**Figure 6** Effect of ganglioside $G_{M3}$ on EGF receptor dephosphorylation in A431 cell membranes. A431 cell membranes were incubated with or without 60 μg ml⁻¹ $G_{M3}$ as indicated and EGF. EGFR autophosphorylation was initiated by addition of [$\gamma^{32}$P]ATP and allowed to proceed for 10 min on ice. Thereafter, the reaction was quenched by addition of EDTA and receptor dephosphorylation was monitored by analysing aliquots corresponding to 10 μg of membrane protein at the indicated time points by SDS-PAGE and autoradiography for the EGF receptor phosphate content. The relative radioactivity in the receptor bands was quantified with a Phosphoimager. (**A**) Autoradiograph; (**B**) time course of receptor dephosphorylation as obtained by Phosphorimager analysis of the relative radioactivity in the EGF receptor band. The depicted experiment is one of three with identical results

*egf/r3* inhibits EGFR autophosphorylation. In comparison with H125 cells, αEGFR *ior egf/r3* alone was less effective, and $G_{M3}$ was more effective in inhibiting EGFR activity in A431 cells. The combined inhibition of receptor autophosphorylation by both agents was more pronounced in A431 than in H125 cells (Figures 4B and 5A).

### $G_{M3}$ activates EGFR-directed PTPase activity

Interestingly, when the A431 cell treatment was performed in the presence of pervanadate, a potent inhibitor of PTPases (Pumiglia et al, 1992), the inhibition of EGFR autophosphorylation by $G_{M3}$ as well as the synergistic inhibition of EGFR autophosphorylation by $G_{M3}$ and αEGFR *ior egf/r3*, was abolished (Figure 5C). Also, $G_{M3}$-mediated inhibition of EGFR autophosphorylation in H125 cells is prevented by pervanadate treatment (Figure 4D). These findings indicate that activation of EGFR-directed PTPases rather than direct tyrosine kinase inhibition by $G_{M3}$ might at least contribute to the observed attenuation of EGFR autophosphorylation. To test directly whether $G_{M3}$ has the capacity to activate EGFR-directed PTPase activity, in vitro PTPase assays employing A431 cell membranes were carried out. Membranes were pretreated with $G_{M3}$ or vehicle, the EGFR was stimulated and autophosphorylation was allowed to occur in the presence of [$\gamma^{32}$P]ATP. The autophosphorylation was quenched by addition of EDTA, and EGFR dephosphorylation by the endogenous PTPases was monitored by analysis of the receptor phosphorylation state at different time points thereafter. As shown in Figure 6, EGFR dephosphorylation is clearly accelerated in the presence of 60 μM $G_{M3}$.

### DISCUSSION

Lung cancer cells, in particular NSCLC, frequently express EGFR. Thus, although the prognostic significance of EGFR expression in lung carcinoma is currently controversial (Modjtahedi and Dean, 1994), the EGFR might present a target for antiproliferative treatment. We compared a limited number of lung carcinoma cells for expression of EGFR by a ligand-binding assay. In accordance with previous observations, five out of six NSCLC cell lines contained measurable levels of EGFR sites per cell, while only one out of five SCLC cell lines was EGFR positive. H125, an NSCLC line with about $2 \times 10^5$ EGF receptors per cell, was chosen to investigate the antiproliferative potency of an EGFR-blocking monclonal antibody (αEGFR *ior egf/r3*) towards NSCLC. Apparently, the growth of this cell line is at least partly dependent on autocrine activation of the EGFR, since: (1) the cells are known to express TGFα (Söderdahl et al, 1988) in addition to EGFR; (2) the cells respond only moderately to exogenous EGF; and (3) most importantly, the cells are growth inhibited by very low doses of highly specific EGFR tyrosine kinase inhibitors, whereas NSCLC cells not expressing EGFR were completely refractory to the drugs. The anti-EGF receptor antibody, αEGFR *ior egf/r3*, reduced the growth of H125 cells under the same culture conditions to 41%, without cytotoxic effect. These results match well with those from ongoing in vivo studies with αEGFR *ior egf/r3*. No relevant toxic effects have been found; however, the cytostatic effects on the tumours observed so far are only moderate (unpublished data). Similar results have been reported for other studies employing EGFR-blocking antibodies (Baselga and Mendelsohn, 1994*b*), suggesting the need to combine this treatment with another antiproliferative principle. We therefore

 © Cancer Research Campaign 1997

investigated the effect of a combined antiproliferative cell treatment with the anti-EGF receptor antibody, αEGFR*ior egf/r3*, and gangliosides, again using the NSCLC line H125 as a target. Indeed, ganglioside $G_{M3}$ (but not De-*N*-acetlyl$G_{M3}$) was found greatly to potentiate the effect of αEGFR *ior egf/r3* on the growth of these EGFR-expressing cells leading to almost complete growth arrest and cytotoxicity. This effect was dependent on the presence of EGFR, since growth of an NSCLC line lacking EGFR expression was completely unaffected by the combined treatment. Synergistic growth inhibition was also observed using another NSCLC cell line (U1752) as target or using the combination of another anti-EGFR antibody (αEGFR *mab425*) and $G_{M3}$ for the treatment, suggesting that the observed synergism is general. Experiments are underway to analyse further this new cytostatic concept in experimental tumours in vivo. A similar synergistic growth inhibition by a combination of an anti-EGFR antibody and a synthetic tyrosine kinase inhibitor has been observed for a squamous cell carcinoma by Yoneda et al (1991).

When we compared the effect of various treatments of H125 cells on cell growth with that on EGFR autophosphorylation activity, we observed an overall correlation of the inhibitory effects. Cell growth was blocked by specific EGFR tyrosine kinase inhibitors, the anti-EGF receptor antibody αEGFR *ior egf/r3* inhibited receptor autophosphorylation and the combination of αEGFR *ior egf/r3* and $G_{M3}$ had a stronger effect on EGFR autophosphorylation than either agent alone. Taken together, these correlations suggest that the observed EGFR tyrosine kinase inhibition is causally related to growth inhibition. Some differences in the dose – response characteristics for growth inhibition and kinase attenuation were, however, observed. Most notably, the combined inhibition by αEGFR *ior egf/r3* and $G_{M3}$ was less pronounced on the level of H125 cell EGFR autophosphorylation than on the level of cell growth. These quantitative differences might be a result of the somewhat different treatment and assay schedules for the two parameters. It is, however, currently not possible to exclude additional effects of the agents used on other cellular systems contributing to the observed growth inhibition.

Growth factor receptor signalling activity at the level of receptor autophosphorylation is the net result of the action of receptor PTK and opposing PTPases. We therefore investigated whether the synergistic inhibitory effect of αEGFR *ior egf/r3* and $G_{M3}$ on the EGFR autophosphorylation involved merely tyrosine kinase inhibition or possibly also effects on the EGFR-directed PTPase(s). Interestingly, the attenuation of EGFR autophosphorylation by $G_{M3}$ in H125 cells and in A431 cells is abrogated by pretreatment of the cells with the PTPase inhibitor, pervanadate. Furthermore, $G_{M3}$ has the capacity to activate EGFR dephosphorylation in A431 cell membranes in vitro. Thus, activation of EGFR-directed PTPases by $G_{M3}$ seems to be involved in the attenuation of EGFR signalling activity, possibly in addition to a direct tyrosine kinase inhibition (Zhou et al, 1994). It seems tempting to speculate that PTPase activation is also likely to be involved in the enhancement of anti-EGFR antibody-mediated growth inhibition by $G_{M3}$ in H125 NSCLC cells. The identity of the activated PTPase(s) is currently unknown. The SH2-domain PTPase 1C has been shown to attenuate EGFR signalling in A431 cells; however, it is unlikely that this cytosolic PTPase is affected by exogenously added $G_{M3}$. Rather transmembrane PTPases (Charbonneau and Tonks, 1992; Pot and Dixon, 1992) are candidate targets for $G_{M3}$ action. One could envisage $G_{M3}$ effects on such PTPases either via the membrane spanning or via the extracellular protein domains. The observed activation by $G_{M3}$ might help to identify further PTPases involved in EGFR dephosphorylation and might present a new regulatory principle for PTPases. Furthermore, our findings lend support to the concept that activation of growth factor receptor-directed PTPases could be employed as a mechanism for novel antiproliferative agents.

### ABBREVIATIONS

EGF, epidermal growth factor; EGFR, epidermal growth factor receptor; EDTA, ethylenediamine tetraacetic acid; FCS, fetal calf serum; DMSO, dimethyl sulphoxide; SCLC, small-cell lung carcinoma; NSCLC, non-small-cell lung carcinoma; SDS, sodium dodecyl sulphate; PAGE, polyacrylamide gel electrophoresis; PTPase, protein tyrosine phosphatase.

### ACKNOWLEDGEMENTS

We thank Drs Gazit and Levitzki for the generous gift of the EGF receptor blockers, AG1478 and AG1517, and Dr Luckenbach for αEGFR *mab425*. We are indebted to Drs Bergh and Gazdar for the generous provision of the lung carcinoma cell lines used in this study. Udo Greiser was supported by a scholarship from DFG Sonderforschungsbereich 197 (Friedrich-Schiller Universität, Jena) and Eduardo Suarez Pestana by a scholarship of DAAD.

### REFERENCES

Barnes DW (1982) Epidermal growth factor inhibits growth of A431 human epidermoid carcinoma in serum free cell culture. *J Cell Biol* **93**: 1–4

Baselga J and Mendelsohn J (1994*a*) The epidermal growth factor receptor as a target for therapy in breast carcinoma. *Breast Cancer Res Treat* **29**: 127–138

Baselga J and Mendelsohn J (1994*b*) Receptor blockade with monoclonal antibodies as anti-cancer therapy. *Pharmacol Ther* **64**: 127–154

Bergh J, Larsson E, Nilsson K and Zech L (1982) Establishment and characterization of two neoplastic cell lines (U-1285 and U-1568) derived from small cell carcinoma of the lung. *Acta Pathol Microbiol Immunol Scand A* **90**: 149–158

Bergh J, Nilsson K, Zech L and Glovanella P (1981) Establishment and characterization of a continuous lung squamous cell carcinoma cell line (U-1752). *Anticancer Res* **1**: 317–322

Bergh J, Nilsson K, Ekman R and Giovanella B (1985) Establishment and characterization of cell lines from human small cell and large cell carcinomas of the lung. *Acta Pathol Microbiol Immunol Scand A* **93**: 133–147

Böhmer FD, Böhmer, SA and Heldin, CH (1993) The dephosphorylation characteristics of the receptors for epidermal growth factor and platelet-derived growth factor in Swiss 3T3-cell membranes suggest differential regulation of receptor signalling by endogenous protein-tyrosine phosphatases. *FEBS Lett* **331**: 276–280

Böhmer FD, Böhmer A, Obermeier A and Ullrich A (1995) Use of selective tyrosine kinase blockers to monitor growth factor receptor dephosphorylation in intact cells. *Anal Biochem* **228**: 267–273

Bremer EG, Schlessinger J and Hakomori SI (1986) Ganglioside-mediated modulation of cells growth. Specific effects of $G_{M3}$ on tyrosine phosphorylation of the epidermal growth factor receptor. *J Biol Chem* **261**: 2434–2440

Carney DN, Gazdar AF, Bepler G, Guccion GJ, Marangos PJ, Moody TW, Zweing MH and Minna JD (1985) Establishment and identification of small cell cancer cell line having classic and variant features. *Cancer Res* **45**: 2913–2923

Charbonneau, H and Tonks NK (1992) 1002 phosphatases? *Annu Rev Cell Biol* **8**: 463–493

Cohen S and Carpenter G (1975) Human epidermal growth factor: isolation and chemical and biological properties. *Proc Natl Acad Sci USA* **72**: 1317–1321

Derynck R, Goeddel DV, Ullrich A, Gutterman JU, Williams RD, Bringman TS and Berger WH (1987) Synthesis of messenger RNAs for transforming growth factors alpha and beta and the epidermal growth factor receptor by human tumors. *Cancer Res* **47**: 707–712

© Cancer Research Campaign 1997 

Di Fiore P, Pierce JH, Fleming TP, Hazan R, Ullrich A, King CR, Schlessinger J and Aaronson SA (1987) Overexpression of the human EGF receptor confers an EGF-dependent transformed phenotype to NIH 3T3 cells. *Cell* **51**: 1063–1070

Downward J, Yarden Y, Mayes E, Scrace G, Totty N, Stockwell P, Ullrich A, Schlessinger J and Waterfield MD (1984) Close similarity of epidermal growth factor receptor and v-*erb-B* oncogene protein sequences. *Nature* **307**: 521–527

Faure R, Baquiran G, Bergeron JJM and Posner BI (1992) The dephosphorylation of insulin and epidermal growth factor receptors. Role of endosome-associated phosphotyrosine phosphatases. *J Biol Chem* **267**: 11215–11221

Fernandez A, Spitzer E, Perez R, Boehmer FD, Eckert K, Zschiesche W and Grosse R (1992) A new monoclonal antibody for detection of EGF-receptors in Western blots and paraffin-embedded tissue sections. *J Cell Biochem* **49**: 157–165

Fong CJ, Sherwood ER, Mendelsohn J, Lee C and Kozlowski JM (1992) Epidermal growth factor receptor monoclonal antibody inhibits constitutive receptor phosphorylation, reduces autonomous growth, and sensitizes androgen-independent prostatic carcinoma cells to tumor necrosis factor-alpha. *Cancer Res* **52**: 5887–5892

Fonseca R, Lombardero J, Perez R, Quintero S, Tormo B, Macias A, Rios M, Cordies N, Fony F, Leon B, Puig M, Aguilar O, Rodriguez C and Lage A (1988) Effecto antitumoral del factor de crecimiento epidermico en carcinoma de piel humano. *Interferon Biotecnologia* **5**: 270–277

Fontanini G, Vignati S, Bigini D, Mussi A, Lucchi H, Angeletti CA, Pingitore R, Pepe S, Basolo F and Bevilacqua G (1995) Epidermal growth factor receptor (EGFr) expression in non-small cell lung carcinomas correlates with metastatic involvement of hilar and mediastinal lymph nodes in the squamous subtype. *Eur J Cancer* **31A**: 178–183

Fry DW, Kraker AJ, Mcmichael A, Ambroso LA, Nelson JM, Leopold WR, Conners RW and Bridges AJ (1994) A specific inhibitor of the epidermal growth factor receptor tyrosine kinase. *Science* **265**: 1093–1095

Gazdar AF, Carney DN, Russel EK, Smis HL, Bylin SB, Bunn JR, Guccion GJ and Minna AJD (1980) Establishment of continous clonable culture of small cell carcinoma of the lung which have amino precursor uptake and decarboxylation cell properties. *Cancer Res* **40**: 3502–3507

Gibbs JB and Oliff A (1994) Pharmaceutical research in molecular oncology. *Cell* **79**: 193–198

Groenen LC, Nice EC, and Burgess AW (1994) Structure–function relationships for the EGF/TGFα family of mitogens. *Growth Factors* **11**: 235–257

Hakomori S (1993) Structure and function of sphingoglycolipids in transmembrane signalling and cell cell interactions. *Biochem Soc Trans* **21**: 583–595

Hanai N, Nores GA, Macleod C, Torres MC and Hakomori S (1988) Ganglioside-mediated modulation of cell growth. Specific effects of $G_{M3}$ and lyso-$G_{M3}$ in tyrosine phosphorylation of the epidermal growth factor receptor. *J Biol Chem* **263**: 10915–10921

Hashimoto N, Zhang WR and Goldstein BJ (1992) Insulin receptor and epidermal growth factor receptor dephosphorylation by three major rat liver protein-tyrosine phosphatases expressed in a recombinant bacterial system. *Biochem J* **284**: 569–576

Hayman MJ, Kitchener G, Vogt PK and Beug H (1985) The putative transforming protein of S13 avian erythroblastosis virus is a transmembrane glycoprotein with an associated protein kinase activity. *Proc Natl Acad Sci USA* **82**: 8237–8241

Kamata N, Chida K, Rikimaru K, Horikoshi M, Enomoto S and Kuroki T (1986) Growth-inhibitory effects of epidermal growth factor and overexpression of its receptors on human squamous cell carcinomas in culture. *Cancer Res* **46**: 1648–1653

Khazaie K, Schirrmacher V and Lichtner RB (1993) EGF receptor in neoplasia and metastasis. *Cancer Metas Rev* **12**: 255–274

Klijn JGM, Berns PMJJ, Schmitz PIM and Foekens JA (1992) The clinical significance of epidermal growth factor receptor (EGFR) in human breast cancer: a review on 5232 patients. *Endocrine Rev* **13**: 3–17

Lammers R, Bossenmaier B, Cool DE, Tonks NK, Schlessinger J, Fischer EH and Ullrich A (1993) Differential activities of protein tyrosine phosphatases in intact cells. *J Biol Chem* **268**: 22456–22462

Levitzki A and Gazit A (1995) Tyrosine kinase inhibition: an approach to drug development. *Science* **267**: 1782–1788

Lombardero J, Perez R and Lage A (1986) Epidermal growth factor inhibits thymidine incorporation in Ehrilich ascites tumor cells *in vivo*. *Neoplasma* **33**: 423–429

Macias A, Azavedo E, Perez R, Rutqvist LE and Skoog L (1986) Receptors for epidermal growth factor in human mammary carcinomas and their metastases. *Anticancer Res* **6**: 849–851

Macias A, Azavedo E, Hagerstrom T, Klintenberg C, Perez R and Skoog L (1987*a*) Prognostic significance of the receptor for epidermal growth factor in human mammary carcinomas. *Anticancer Res* **7**: 459–464

Macias A, Perez R, Hagerstrom T and Skoog L (1987*b*) Identification of transforming growth factor alpha in human primary breast carcinomas. *Anticancer Res* **7**: 1271–1275

Modjtahedi H and Dean C (1994) The receptor for EGF and its ligands – expression, prognostic value and target for therapy in cancer (review). *Int J Oncol* **4**: 277–296

Modjtahedi H, Eccles S, Box G, Styles J and Dean C (1993) Immunotherapy of human tumour xenografts overexpressing the EGF receptor with rat antibodies that block growth factor – receptor interaction. *Br J Cancer* **67**: 254–261

Mueller B, Romerdahl CA, Trent JM and Reisfeld RA (1991) Suppression of spontaneous melanoma metastasis in SCID mice with an antibody to the epidermal growth factor receptor. *Cancer Res* **51**: 2193–2198

Nores GA, Hanai N, Levery SB, Eaton HL, Salyan ME and Hakomori S (1989) Synthesis and characterization of ganglioside GM3 derivatives: lyso-GM3, de-*N*-acetyl-GM3, and other compounds. *Methods Enzymol* **179**: 242–253

Osherov N and Levitzki A (1994) Epidermal-growth-factor-dependent activation of the Src-family kinases. *Eur J Biochem* **225**: 1047–1053

Perez R, Pascual M, Macias A and Lage A (1984) Epidermal growth factor receptors in human breast cancer. *Breast Cancer Res Treat* **4**: 189–193

Pot DA and Dixon JE (1992) A 1000 and 2 protein tyrosine phosphatases. *Biochim Biophys Acta* **1136**: 35–43

Pumiglia KM, Lau LF, Huang CK, Burroughs S and Feinstein, MB (1992) Activation of signal transduction in platelets by the tyrosine phosphatase inhibitor pervanadate (vanadyl hydroperoxide). *Biochem J* **286**: 441–449

Reins HA, Steinhilber G, Freiberg B and Anderer FA (1993) Anti-epidermal growth factor receptor monoclonal antibodies affecting signal transduction. *J Cell Biochem* **51**: 236–248

Rodeck U, Herlyn M, Herlyn D, Molthoff C, Atkinson B, Varello M, Steplewski, Z and Koprowski, H (1987) Tumor growth modulation by a monoclonal antibody to the epidermal growth factor receptor: immunologically mediated and effector cell-independent effects. *Cancer Res* **47**: 3692–3696

Sainsbury JR, Farndon JR, Sherbet GV and Harris AL (1985) Epidermal-growth-factor receptors and oestrogen receptors in human breast cancer. *Lancet* **1**: 364–366

Scatchard G (1949) The attraction of protein for small molecules and ions. *Ann N Y Acad Sci* **5**: 660–665

Söderdahl G, Betzholz C, Johansson A, Nilsson K and Bergh J (1988) Differential expression of platelet-derived growth factor and transforming growth factor genes in small-and non-small-cell human lung carcinoma lines. *Int J Cancer* **41**: 636–641

Svennerholm L (1973) Gangliosides, isolation. *Methods Carbohydr Chem* **6**: 430–603

Svennerholm L (1994) Gangliosides – a new therapeutic agent against stroke and Alzheimer's disease. *Life Sci* **55**: 2125–2134

Swarup G, Cohen S and Garbers DL (1982) Inhibition of membrane phosphotyrosyl-protein phosphatase activity by vanadate. *Biochem Biophys Res Comm* **107**: 1104–1109

Tomic S, Greiser U, Lammers R, Kharitonenkov A, Imyanitov E, Ullrich A and Böhmer FD (1995) Association of SH2 domain protein tyrosine phosphatases with the epidermal growth factor in human tumor cells. Phosphatidic acid activates receptor dephosphorylation by PTP1C. *J Biol Chem* **270**: 21277–21284

Ullrich A, and Schlessinger J (1990) Signal transduction by receptors with tyrosine kinase activity. *Cell* **61**: 203–212

Velu TJ, Beguinot L, Vass WC, Willingham MC, Merlino GT, Pastan I and Lowy, DR (1987) Epidermal-growth-factor-dependent transformation by a human EGF receptor protooncogene. *Science* **238**: 1408–1410

Weis FM and Davis RJ (1990) Regulation of epidermal growth factor receptor signal transduction. Role of gangliosides. *J Biol Chem* **265**: 12059–12066

Yoneda T, Lyall RM, Alsina MM, Persons PE, Spada AP, Levitzki A, Zilberstein A and Mundy GR (1991) The antiproliferative effects of tyrosine kinase inhibitors tyrphostins on a human squamous cell carcinoma *in vitro* and in nude mice. *Cancer Res* **51**: 4430–4435

Zhou QG Hakomori S Kitamura K and Igarashi Y (1994) G(M3) directly inhibits tyrosine phosphorylation and de-*N*-acetyl-G(M3) directly enhances serine phosphorylation of epidermal growth factor receptor, independently of receptor–receptor interaction. *J Biol Chem* **269**: 1959–1965

© Cancer Research Campaign 1997